# The psychosocial cost burden of cancer: A systematic literature review

**DOI:** 10.1002/pon.5516

**Published:** 2020-09-06

**Authors:** Beverley M. Essue, Nicolas Iragorri, Natalie Fitzgerald, Claire de Oliveira

**Affiliations:** ^1^ Health Economics and Organization Performance The Canadian Partnership Against Cancer Toronto Ontario Canada; ^2^ Institute of Health Policy Management and Evaluation University of Toronto Toronto Ontario Canada; ^3^ University of York York England

**Keywords:** cancer, economic burden, psycho‐oncology, psychosocial costs, quality of life, systematic review

## Abstract

**Background and Objective:**

Psychosocial costs, or quality of life costs, account for psychological distress, pain, suffering and other negative experiences associated with cancer. They contribute to the overall economic burden of cancer that patients experience. But this category of costs remains poorly understood. This hinders opportunities to make the best cancer control policy decisions. This study explored the psychosocial cost burden associated with cancer, how studies measure psychosocial costs and the impact of this burden.

**Methods:**

A systematic literature review of academic and grey literature published from 2008 to 2018 was conducted by searching electronic databases, guided by the Institute of Medicine’s conceptualization of psychosocial burden. Results were analyzed using a narrative synthesis and a weighted proportion of populations affected was calculated. Study quality was assessed using the Ottawa‐Newcastle instrument.

**Results:**

A total of 25 studies were included. There was variation in how psychosocial costs were conceptualized and an inconsistent approach to measurement. Most studies measured social dimensions and focused on the financial consequences of paying for care. Fewer studies assessed costs associated with the other domains of this burden, including psychological, physical, and spiritual dimensions. Fourty‐four percent of cancer populations studied were impacted by psychosocial costs and this varied by disease site (38%‐71%). Two studies monetized the psychosocial cost burden, estimating a lifetime cost per case ranging from CAD$427753 to CAD$528769. Studies were of varying quality; 60% of cross‐sectional studies had a high risk of bias.

**Conclusions:**

Consistency in approach to measurement would help to elevate this issue for researchers and decision makers. At two‐thirds of the total economic burden of cancer, economic evaluations should account for psychosocial costs to better inform decision**‐**making. More support is needed to address the psychosocial cost burden faced by patients and their families.

## 1 INTRODUCTION

The rising costs of cancer treatment and supportive care^1^ are a concern for health care systems, patients, and their families. Due to the large economic burden of cancer care, it is important to have an accurate estimate of the costs associated with cancer and a good understanding of who bears those costs.^2^ Cost‐of‐illness studies can help translate the adverse effects of diseases into dollars, which is one input to support decision**‐**making. This information is crucial to help set future health budgets, to help aid in the allocation of scarce resources and, ultimately, supports decision‐making for cancer control systems.

**FIGURE 1:**
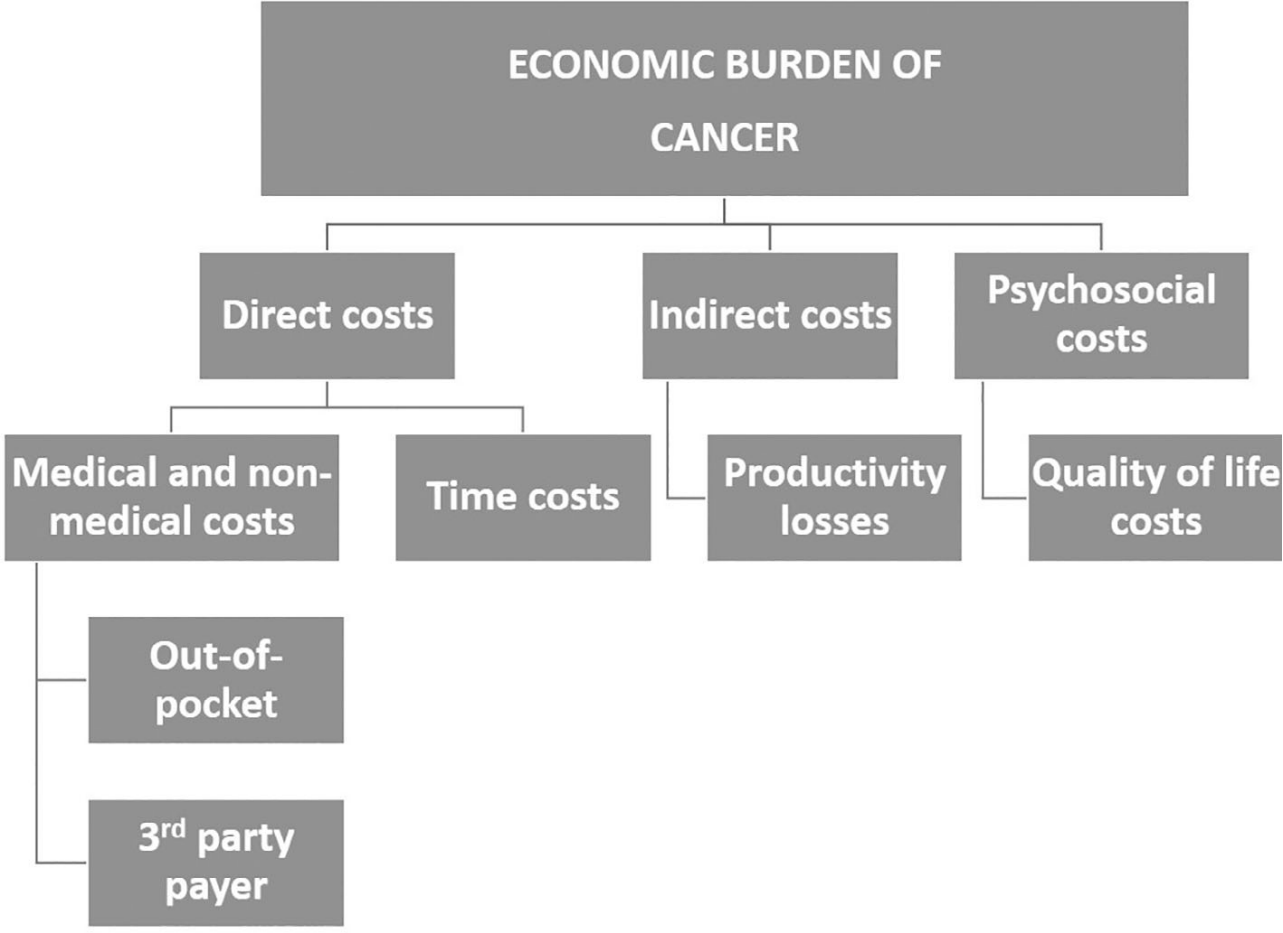
Contributors to the economic burden of cancer (from ^3^)

Generally, the economic burden of cancer care has been described as three broad categories: direct costs, indirect costs, and psychosocial costs^3^ (Figure 1). Direct costs are those that include the use of resources for medical and non‐medical care as well the time spent obtaining such care. Indirect costs are those that result from the loss of resources and opportunities due to cancer. Psychosocial costs have generally been defined as intangible costs associated with cancer, such as pain and suffering.^4^ Psychosocial costs are entirely borne by patients and their families and are the least well understood category of the three.

**FIGURE 2:**
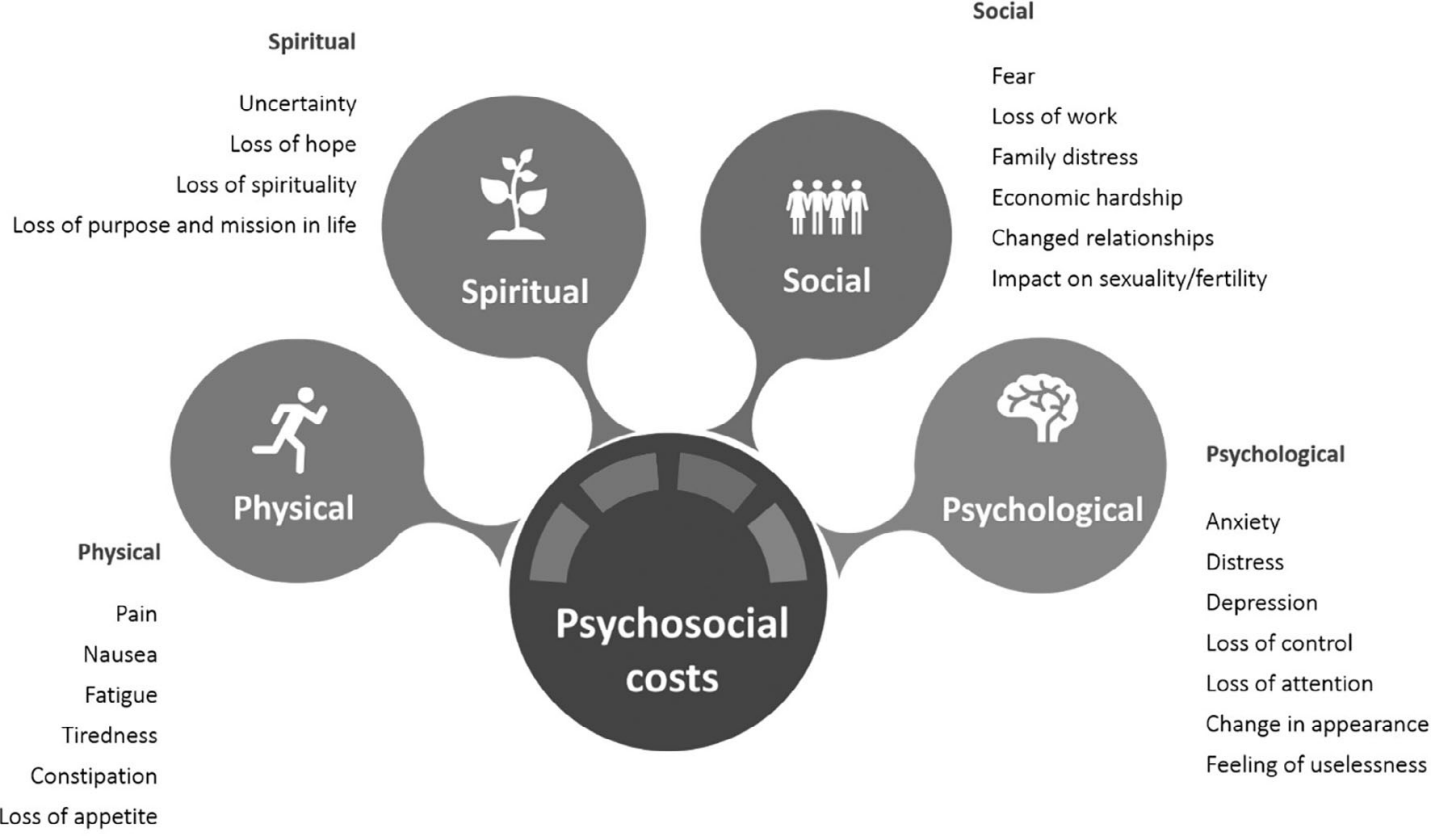
Dimensions of the psychosocial burden of cancer (adapted from ^9^)

The psychosocial burden of cancer refers to the myriad ways that cancer and the cancer experience impact patients and their families, including caregivers. This burden is conceptualized as resulting from impacts on four domains: physical, psychological, spiritual, and social well‐being (Figure 2). A recent study found most cancer survivors in Canada face ongoing and unmet needs related to psychological (90%), physical (80%), and practical (50%) challenges.^5^ These challenges and their collective burden can affect the quality of life of patients and family members involved in their care.^5^ For this reason, psychosocial costs or the psychosocial cost burden have been described as synonymous with quality of life costs.^3^, ^6^

Psychosocial costs represent the additional cost to individuals’ well‐being that is associated with cancer. A seminal definition describes psychosocial costs as follows:

Illness and disease are responsible for a wide variety of deteriorations in quality of life that are frequently referred to as psychosocial costs… Disease may bring about personal catastrophes that are not reflected in the direct and indirect economic costs that are usually estimated for a specific disease… These include, but are not limited to, undesired changes in life plans, anxiety, reduced self‐esteem and feeling of well‐being, and other emotional problems… psychosocial costs are a significant, and very likely quite large, component of the total burden of illness. To ignore them, or misrepresent them, can result in an underestimate of the impact of disease and bias the decision‐making process.^7^

In other literature, psychosocial costs have been described as an intangible cost due to the challenges of assigning a monetary value.^8^ These costs are distinct from, and in addition to, the direct costs that may be paid for using additional health care services to address psychosocial issues, for example, to see a psychologist. Psychosocial costs are also related to, but distinct, from the indirect costs borne by society due to lost productivity that may result from an unresolved symptom burden during treatment that prevents a patient from working.

These challenges with the definition of psychosocial costs have resulted in a mixed approach to measurement. Some studies approach measurement of these costs using generic assessments of quality of life that may not capture the full realm of psychosocial burden according to the four key domains, namely: physical, psychological, spiritual, and social well‐being. While some of this impact may in fact be captured in generic quality of life assessments, it is unclear whether and to what extend the generic tools are able to capture the full breadth and impact of the psychosocial burden of illness. There is also a varied approach to measurement of psychosocial costs. The inconsistent approach to examining these costs in the literature creates missed opportunities for leveraging these learnings to inform decision‐making and resource allocation. Furthermore, despite recognition of the additional burden caused by psychosocial costs raised in the Hosdgeson and Meiners 1982 paper, the methods to advance to measurement and use of psychosocial cost data have been scare, particular in the health economics literature.

Understanding the scope and scale of the cancer burden borne by patients and families is important to properly and accurately measure the overall economic burden of cancer to society. It is also required if we are to take seriously our commitments and value placed on patient‐centered care within the cancer control system. Moreover, this information is necessary to inform decision‐making around best models of care to support patients and the patient experience throughout the cancer trajectory. It is also critical for monitoring the potential impact—even inadvertent—of shifts in cost out of the system that may result in an economic burden for patients.

The objective to this review was to answer the following research questions: (a) what is the psychosocial cost burden associated with cancer and how do studies measure psychosocial costs? and (b) what is the prevalence and impact of this burden?

## 2 METHODS

### 2.1 Search strategy, selection criteria, and retrieval of articles

The review was registered on PROSPERO CRD42019133975.

In this review, we drew on the definition of psychosocial burden from the Institute of Medicine’s conceptual framework. We aimed to understand and describe the estimated costs associated with this burden, either in monetary terms or when defined as intangible costs. Psychosocial consequences of cancer can constitute a cost to patients and caregivers that may or may not have been monetized. These consequences also have potential to lead to direct economic impacts for patients and their families, which also may or may not be monetized. This review intentionally casts its scope broadly to capture all these potential conceptualizations of psychosocial costs, aligned with the definition of psychosocial costs provided by Hodgson 1982.^7^

A detailed search strategy was developed and informed by the Institute of Medicine’s conceptual framework for the psychosocial burden of cancer,^9^ with input from a medical librarian and content experts (see online supplement in Data S1). This framework was used as it reflects the general consensus on the main features of the psychosocial burden associated with cancer and we were interested in understanding all of the associated costs. After piloting and adjusting the search strategy, we searched the following databases: Pubmed, Medline, Embase, CINAHL, PsycInfo, Econlit, and the Johanna Briggs EBP database. Google Scholar was searched using keywords from the main search strategy. In addition, grey literature was searched using a modified search strategy in OpenGrey and Grey Literature Report. The reference lists of all included papers were reviewed to identify potentially relevant papers. We also consulted experts to identify other relevant studies. Only studies in English were considered.

We aimed to include all evidence on the psychosocial cost burden associated with cancer in all populations. We included pediatric and adult cancers to understand whether there were important differences in the cost estimates, the approaches to measurement used across studies as well as the extent to which this issue was measured across different patient populations. For pediatric cancers, the caregiver’s/parent’s costs, reflect a main source of economic burden associated with these types of cancers. In addition, and importantly, patient and caregiver costs are ultimately borne and shared at the household‐level. For these reasons, both adult and pediatric populations were considered within scope of this review.

The titles and abstracts of all retrieved papers were independently screened for eligibility by two individuals and, subsequently, the full text articles of the remaining articles, using pre‐specified inclusion criteria. Articles were included if they were published between the years 2008 and 2018, were a primary research study that focused on measuring or describing the psychosocial cost burden associated with cancer, and were conducted in a population of patients at any point along the cancer care continuum (from diagnosis to palliative and end‐of‐life care, including survivorship). Articles were excluded if they were not written in English. Consistency in the screening process between individuals was ensured by having each person independently review between 20 and 50 papers and then compare their results with each other, discussing and resolving any discrepancies and consulting with the research team as needed.

A form was developed to guide the data extraction process. The form captured information on study characteristics (eg, jurisdiction, setting, design, data sources, or tools used) measurement/definition of outcomes, key demographic characteristics of the research population (eg, age, proportion female, cancer site, point of care along the cancer care continuum, perspective), dimensions of the psychosocial burden (physical, spiritual, social, and psychological) and its measurement, and key findings.

We assessed the risk of bias in the cross‐sectional and cohort studies using the Ottawa‐Newcastle quality appraisal tool.^10^ The tool assessed study quality using a “star system,” from three broad perspectives: the selection of the study population; the comparability of the study population; and the ascertainment of either the exposure or outcome of interest.

The results were first analyzed using a narrative synthesis that focused on distilling the main dimensions of the psychosocial cost burden that were the focus of the studies, how they were measured, the factors that contributed to the psychosocial cost burden, and notable differences in this burden between cancer populations. Studies were categorized according to how they conceptualized the psychosocial costs burden, including costs associated with physical, spiritual, social, and psychological well‐being (Figure 2). Subsequently, all estimates of the proportion of the study population who experienced a psychosocial cost burden, regardless of how measured, were synthesized and a weighted average of the proportion and 95% confidence intervals were calculated for each cancer type, by study population (adult and pediatric) and for all studies. The purpose of the weighted average was to describe how prevalent the psychosocial cost burden was across the published studies. Given the heterogeneity in how the costs were estimated, cost data were not synthesized.

This systematic review is reported in accordance with the Preferred Reporting Items for Systematic Reviews and Meta‐analyses (PRISMA) guidelines (online appendix). This was a systematic review, so ethics approval was not required.

## 3 RESULTS

### 3.1 Summary of included studies

**FIGURE 3:**
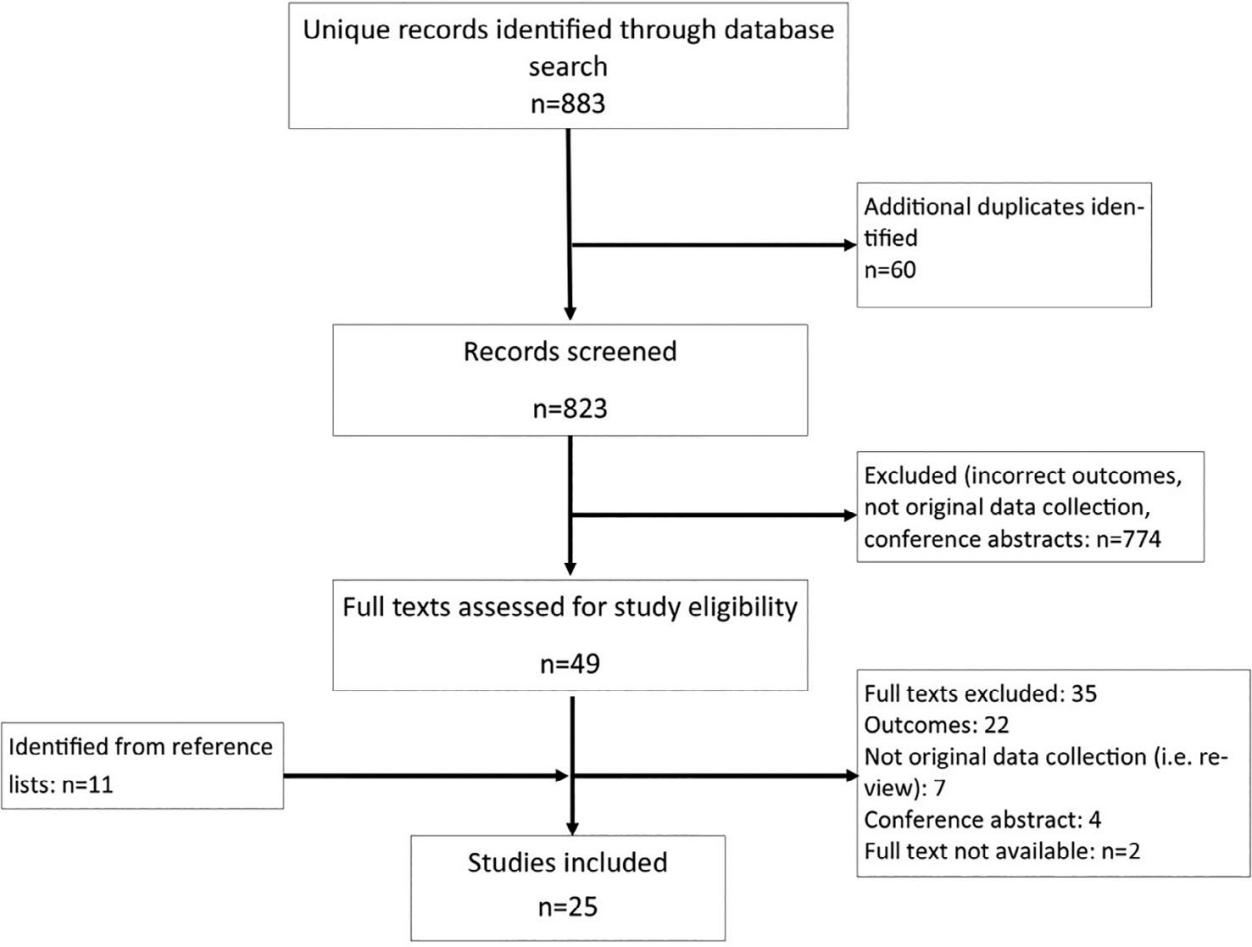
PRISMA Flow Chart of Included Studies

**FIGURE 4:**
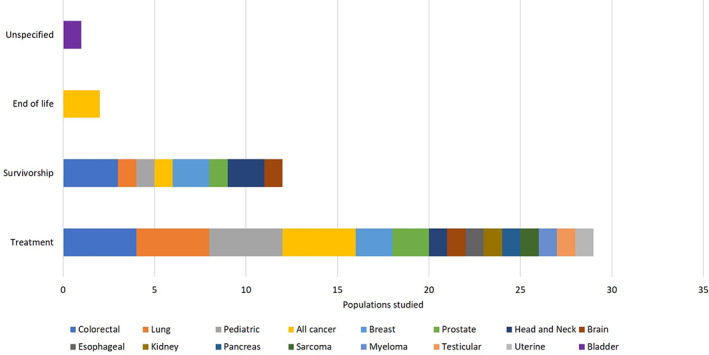
Summary of studies according to cancer care continuum

There were 883 studies retrieved in the search of the academic databases; an additional 11 papers were identified from references lists. No relevant studies were found in the search of the grey literature. After screening and full text review, 25 papers met the inclusion criteria and were included for analysis^6^, ^11^, ^12^, ^13^, ^14^, ^15^, ^16^, ^17^, ^18^, ^19^, ^20^, ^21^, ^22^, ^23^, ^24^, ^25^, ^26^, ^27^, ^28^, ^29^, ^30^, ^31^, ^32^, ^33^, ^34^ (Figure 3). A summary of the included studies is shown in Table 1. The studies reflected a total of 11 331 individuals. Most studies were conducted in adult populations (88%) and recruited from clinical settings (64%) in the United States (84%); two studies were conducted in Canada and two in Ireland. Most studies investigated the psychosocial cost burden associated with multiple cancers, covering the following disease sites: colon/rectum (17.1%), lung (11.4%), breast (11.4%), prostate (8.6%), head, and neck (5.7%); one study each was conducted in populations with cancer of the esophagus, brain, kidney, pancreas, testes, uterus, and bladder. There was also one study each conducted in populations with sarcomas and multiple myeloma. Most of the study populations were undergoing treatment (64%) or were cancer survivors (27%) (Figure 4).

**TABLE 1:** Summary of studies included in the review (n = 25)

Studies	Cancer site; cancer continuum stage	Design; Setting; n	Psychosocial dimension				Proportion of study population affected % (95%CI)	Cost estimate?	Key findings
			Physical	Spiritual	Social	Psychological			
Bestvina et al 2014, USA	Breast, esophageal, kidney, lung, CRC pancreas, prostate, sarcoma, testicular, uterine (69%: breast, lung, CRC, prostate); Treatment	Cross‐sectional; Clinical setting; 300			✓		16 (12,21)	No	Financial distress increases the odds of non‐adherence to treatment.
Bona et al 2014, USA	All cancers (pediatric); Treatment	Cross‐sectional; Clinical setting; 71			✓		27 (15,31)	No	The distribution of the financial burden associated with cancer is inequitable, poorer families reporting disproportionate income losses and impact.
Bona et al. 2016, USA	All cancers (pediatric); Treatment	Cross‐sectional; Clinical setting; 99			✓		29 (18,40)	No	Low‐income and household material hardship (HMH) are prevalent in a significant proportion of newly diagnosed pediatric oncology families despite psychosocial supports available. HMH is a quantifiable and remediable measure of poverty.
Cagle et al. 2016, USA	All cancers; End of life	Cross‐sectional; Community; 176			✓		51 (41,62)	No	The cost of care was a major financial burden for most patients and a third indicated using all or most of their savings. Households with younger or minority (ie, other race) cancer patients are especially at‐risk for financial burden. Financial stress and strain were linked to multiple elements of the cancer care experience, they are related concepts (*r* = 0.46, *P* < .01), but are not synonymous.
Chongpison et al 2016, USA	Rectal; Survivorship	Cross‐sectional; Insured community; 576	✓		✓	✓	22 (18,26)	No	Depression was associated with greater perception of financial burden. Screening for depression and assessing financial well‐being might improve care among long‐term survivors
Creswell et al 2014, USA	All cancers (pediatric); Treatment, survivorship	Case–control; Clinical setting and community; 215			✓	✓	35 (21,48)	No	Financial difficulties have a strong independent association with depressive symptoms among the parents of pediatric patients.
de Souza et al 2016, USA	All cancers; Treatment, end‐of‐life	Cross‐sectional; Clinical setting; 233			✓	✓	nr	No	The COST measure demonstrated reliability and validity in measuring financial toxicity. Its correlation with HRQOL indicates that financial toxicity is a clinically relevant patient‐centered outcome.
Fletcher et al. 2010, USA	All cancers (pediatric); Treatment	Qualitative; Clinical setting; 9			✓		nr	No	Four main costs to parents resulting from children’s cancer: financial and work issues; health of the mothers and other family members; and upheaval of family life.
Huntington et al. 2015, USA	Multiple Myeloma; Treatment	Cross‐sectional; Clinical setting; 100			✓		71 (54,87)	No	Financial toxicity and use of coping mechanisms were common in insured cancer populations.
Jung et al 2018, Canada	Bladder; All stages	Economic evaluation; Model; 199	✓		✓	✓	Nr	Yes—$428 689 per case	At $50 000 per quality adjusted life year, quality of life costs account for 65% of the total lifetime cost of cancer. This accounts for the impact on health of impaired social role engagement, pain, suffering, and loss of employment
Kale et al 2016, USA	All cancers (33%: breast, CRC, prostate); Survivorship	Cross‐sectional; Community; 1380	✓		✓	✓	31 (28,34)	No	Cancer‐related financial burden was associated with lower health‐related quality of life, increased risk of depressed mood, and a higher frequency of worrying about cancer recurrence among survivors
Landwehr et al 2016, USA	All cancers; Survivorship	Cross‐sectional; Grantee database; 371			✓		nr	No	Financial hardship is associated with medical non‐adherence, which affects outcomes. It impacts younger adults more than older adults.
Litzelman et al. 2011, USA	Pediatric (brain); Treatment and survivorship	Cross‐sectional; Clinical setting; 75			✓	✓	71 (52,90)	No	The caregiver burden and financial stress mediate the effect of caring for a child with cancer and impact parental mental health‐related QOL
Massa et al 2018, USA	Head and neck; Treatment and survivorship	Cross‐sectional; Clinical setting; 100			✓		64 (48,80)—financial stress, tests 18 (10,26)—stress, lost income 24 (14,34)—stress 27 (17,37)—stress, transport 31 (21,42)—stress, tests	No	Patients with limited social support, high financial stress, functional deficits, and those with transportation burdens have greater demands for care.
Meisenberg et al 2015, USA	All cancer (42%: breast, CRC, lung); Treatment	Cross‐sectional; Clinical setting; 132			✓		47 (35,59)	No	Financial stress was measured as a side effect of the OOP cost burden associated with cancer
Meneses et al. 2012, USA	Breast; Survivorship	Prospective cohort; Clinical trial; 132			✓		71 (54,87)	No	>50% of survivors reported at least one economic burden event related to either work or financial hardship. More than a quarter reported changes in income or sacrificing family plans. Among those who worked, >15% reported changes in motivation, productivity or quantity (missed days) of work. These events, in turn, were negatively associated with financial burden
O’Brien et al 2016, Ireland	Head and neck, Survivorship	Cross‐sectional; Clinical setting; 583			✓	✓	47 (41,53)	No	Psychological unmet needs comprise seven out of the 10 top needs in this sample
Regenbogen et al. 2014, USA	Colorectal; Post‐operative	Cross‐sectional Clinical setting; 937			✓		71 (66,76)	No	Significantly greater personal financial burden among patients who with postoperative complications, addition, longer time off work and more likely to never resume working. Coping strategies included cutting back on spending for food, clothes, and recreation and to accumulate debt or not pay bills. High levels of worry about finances.
Shankaran et al. USA	Colorectal; Treatment	Cross‐sectional Registry; 284			✓		38 (31,45)	No	Patients at greatest risk for financial hardship and non‐adherence were younger; had lower annual household incomes; or were unemployed, on work disability, or on a leave‐of‐absence.
Sharp et al 2012 Ireland	Breast, lung, prostate; Treatment	Cross‐sectional Clinical setting; 654			✓	✓	32 (28,36) – Financial strain 49 (44,54) – Financial stress	No	Depression risk was raised three‐fold in those reporting cancer‐related financial stress (OR: 2.79)
Tompa et al. 2017, Canada	Lung; Treatment	Cost‐of‐illness; Model; 2331	✓			✓	nr	Yes—Mesothelioma: C$528769/case Lung cancer: C$446288/case	At $50 000 per quality‐adjusted life year, 67.6% of the total lifetime cancer cost is attributed to quality‐of life costs. This accounts for the intrinsic value of health and value for social role engagement.
Veenstra et al. 2014, USA	Colorectal; Post‐operative	Cross‐sectional Registry; 956			✓		62 (57,67)	No	New tool developed and validated to identify patients at risk for financial burden and inform policy interventions. There was substantial financial burden among most respondents, with significantly higher burden reported for those on chemotherapy.
Zafar et al. 2015, USA	Colorectal and lung; Treatment and survivorship	Cross‐sectional Clinical setting; 1000			✓		48 (44,53)	No	Financial burden is prevalent among cancer survivors and is associated with patients’ health‐related quality of life, even after adjusting for the effects of income, employment, disease status and comorbidities
Zafar et al. 2013, USA	All cancers 83%: breast, CRC, lung); Treatment	Cross‐sectional Clinical setting; 254			✓		42 (34,54)	No	Participants struggling to pay for their cancer treatment altered their lifestyles considerably to defray out‐of‐pocket expenses. Copayment assistance applicants were more likely than non‐applicants to employ at least one lifestyle‐altering strategy to cope with costs.
Zullig et al 2013, USA	All cancers, Treatment	Cross‐sectional Community; 164			✓		nr	No	In isolation, subjective financial distress was associated with medication non‐adherence. However, in multivariable analysis, the role of financial distress as a predictor of non‐adherence was less clear

*Note:* CRC: colorectal cancer; nr: not reported; HRQOL, health‐related quality of life. Cancer continuum stage includes the following: diagnosis, treatment, survivorship, and end‐of‐life care.

The Ottawa‐Newcastle tool was used to assess the risk of bias in cross‐sectional studies (20/26 studies) (Figure S1‐online supplement in Data S1). There was a high risk of selection bias in 95% of the studies due to the recruitment of convenience or non‐representative samples. There was also a high risk of bias related to the measurement of outcomes in 76% of studies, as most studies did not conduct a blinded measurement of outcomes or use a gold standard or validated tool. However, most studies completed appropriate analyses that adjusted for potential confounding variables, resulting in 75% of studies being assessed as having a low risk of bias in the comparability of results. Taken together, the overall quality of the evidence in this review was assessed as being low. However, as the focus of this study was on understanding the state of the literature on this topic, the evidence is still deemed to provide useful insights to address the main research questions.

### 3.2 What is the psychosocial cost burden associated with cancer and how is it measured?

There were 24 of 25 studies that focused on the social dimension of the psychosocial burden of cancer. The focus of these studies was on measuring the prevalence of negative financial consequences or impacts associated with the private (ie, out‐of‐pocket) costs of cancer. There was inconsistency in the terminology, definitions, and measurement of outcomes related to financial consequences associated with cancer. Most studies measured the negative consequences to patients and families in terms of the *financial burden* (n = 10), *financial strain or stress* (n = 4), or *financial toxicity* (n = 3) reported by patients and their families. There was also wide variation in the tools used to measure these outcomes, with no gold standard tool acknowledged and few validated tools used to measure the outcomes (Table 2). The cost to patients due to social support needs or poor social functioning was also investigated in a minority of studies.

**TABLE 2:** Outcomes and tools used to measure the psychosocial cost burden of cancer

Psychosocial domain	Outcomes measured	Tool	Validated tool?
Physical (n = 4)	Quality of life (n = 4)	SF‐12	Yes
		City of Hope Quality of Life Colorectal Cancer	No
		Quality of life ‐ breast cancer survivors	No
		Functional Assessment of Cancer Therapy: General (FACT‐G) questionnaire	Yes
Spiritual (n = 0)	‐	‐	
Social (n = 25)	Financial distress (n = 1)	InCharge Financial Distress/Financial Well‐Being Scale	Yes
	Family financial hardship (n = 1)	Survey about Caring for Children with Cancer	No
	Household material hardship (n = 1)	Author developed	No
	Financial strain and stress (n = 4)	Author developed	No
		Depression Anxiety Stress Scales	Yes
		Life Events Questionnaire	No
	Financial burden (n = 10)	Author developed	No
		Breast Cancer Finances Survey	No
	Financial toxicity (n = 3)	COST survey	Yes
		Personal Financial Wellness Scale	Yes
	Financial Challenges (n = 1)	Author developed	No
	Social support (n = 3)	The Duke Social Support and Stress Scale	Yes
		Supportive Care Needs Survey	No
		Oslo‐3 social support scale	Yes
	Social functioning (n = 1)	HUI‐3	Yes
Psychological (n = 9)	Psychological distress or impairment (eg, anxiety, depression) (n = 4)	SF‐12	Yes
		City of Hope Quality of Life Colorectal Cancer	No
		The Center for Epidemiologic Studies Depression Scale	Yes
		National Comprehensive Cancer Network Distress Thermometer	Yes
		Quality of life ‐ breast cancer survivors	No
		Patient reported outcomes	Yes
		Depression Anxiety Stress Scales	Yes
	Stress (n = 1)	Calgary Symptoms of Stress Inventory	Yes

*Note:* Number of studies do not total 25 as some studies measured multiple dimensions of the psychosocial cost burden and used multiple tools.

The burden on patients associated with psychological and physical consequences of cancer and their impact on patients’ well‐being was investigated in six studies and were described in terms of quality of life costs. In these studies, quality of life costs were explicitly conceptualized as the cost to patients of lost opportunities for social engagement reduced social functioning and the impact on their intrinsic value of health.^6^, ^20^ They were also measured as the cost to individuals due to pain, suffering, and loss of enjoyment associated with poor health due to cancer.^20^ Two instruments that were used to quantify quality of life costs included the SF‐12^35^ and the Health Utilities Index (HUI)‐3,^36^ both of which are validated instruments for measuring health‐related quality of life.

A minority of studies (n = 3) focused on the impact of psychological distress and disorders such as stress, anxiety, and depression and their relationship with negative financial outcomes. These studies conceptualized the psychosocial cost burden in terms of the increased risk of experiencing negative outcomes associated with declines in one’s psychological well‐being. Different tools were used in each study, none of which was validated (Table 2).

There were no studies that measured the psychosocial cost burden of cancer in a holistic way, accounting for all four of the dimensions, and none of the studies explicitly measured the costs associated with the spiritual dimension of this burden.

### 3.3 What is the prevalence and impact of the psychosocial cost burden?

The proportion of the study populations reporting psychosocial costs associated with cancer varied between studies, from 16% of patients reporting financial distress in a study of individuals with breast, esophageal, kidney, lung, pancreatic, prostate, sarcoma, testicular, or uterine cancers^11^ to as high as 71% of patients reporting either a significant financial burden or financial toxicity associated with treatment for colorectal cancer^28^ and multiple myeloma.^19^ There was a lower prevalence of this cost burden found in the studies of pediatric populations, ranging from 27%^12^ to 35%^16^ of study populations affected. As most studies were conducted in the United States, where the majority of individuals are not covered under a publicly funded health insurance system and often face significant out‐of‐pocket costs associated with cancer, we looked at the prevalence of the cost burden in non‐US studies and found the estimates ranged between 32%^30^ and 47%^27^ for the studies conducted in Ireland. The prevalence of this cost burden was not estimated in the two studies conducted in Canada.

**FIGURE 5:**
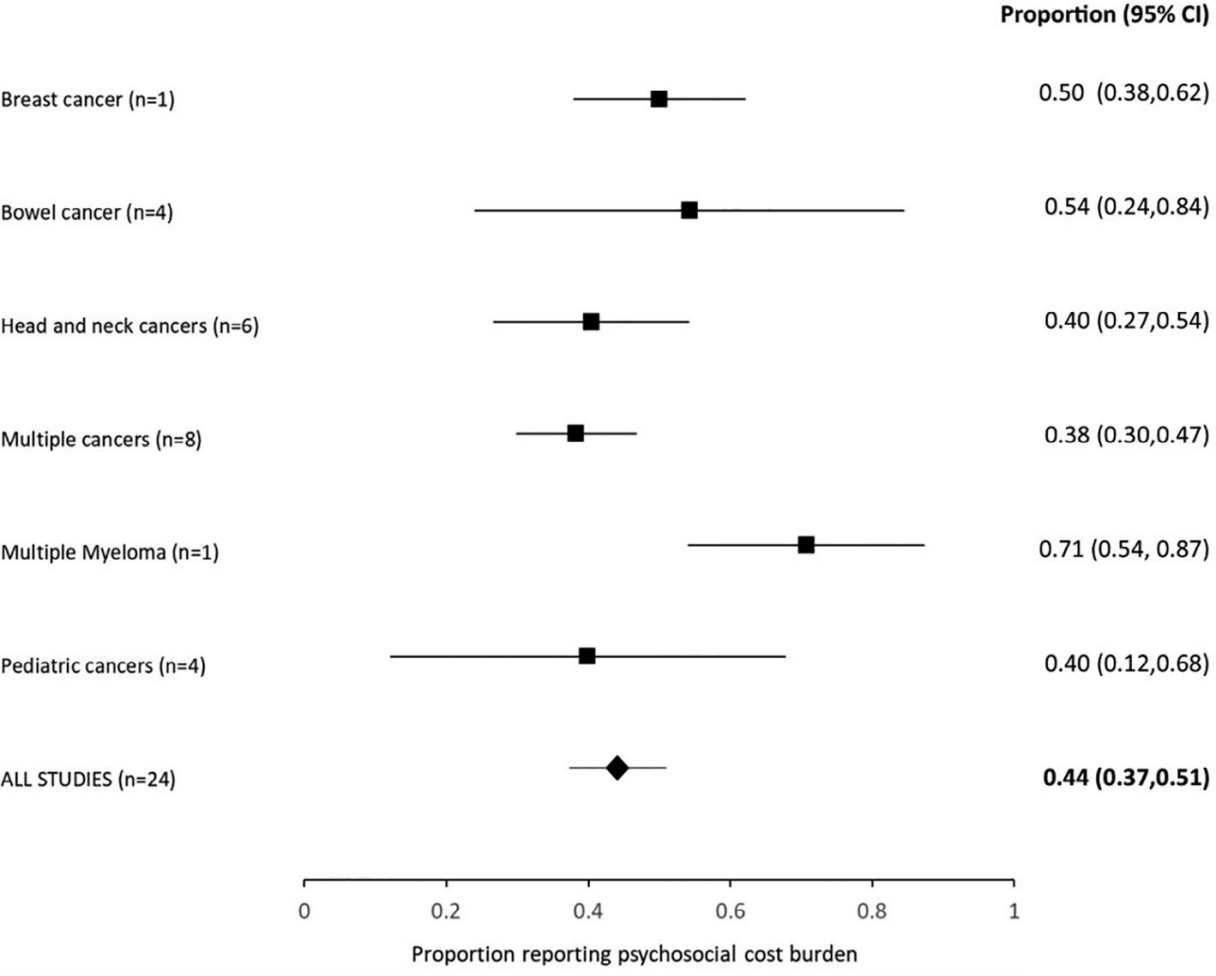
Summary estimates

Figure 5 shows the results of the weighted estimates of the prevalence of the psychosocial cost burden by cancer type. The highest prevalence was found in study populations with multiple myeloma (71%, 95% CI: 54%‐87%), and the lowest estimate was found in studies that included multiple cancers types but predominately focused on breast, colorectal, lung, and prostate cancers (38%, 95% CI: 30%‐47%). The weighted average for the prevalence of the psychosocial cost burden across all studies was 44% (95% CI: 37%‐51%). As most studies focused on measuring the impact on social well‐being and on the financial impact of cancer, this estimate should be interpreted as approximately 44% of cancer patients reported experiencing a financial or economic burden.

Several studies found this burden was associated with an increased risk of facing negative outcomes including: treatment and medication non‐adherence,^11^, ^34^ symptoms of depression,^16^, ^30^ psychological distress,^21^ declines in quality of life^33^ and unmet physical, psychological, sexual, and health system information needs.^27^

Two modeling studies estimated the cost of the psychosocial burden associated with cancer as health‐related quality of life costs, measured using the SF‐12 and the HUI.^6^, ^20^ Both studies approached this estimation by calculating the difference in QALYs between cancer and non‐cancer populations and assigned monetary values for the cancer‐attributable QALYs between CAD$25000 and CAD$100000, informed by the contingent‐value literature.^6^ The lifetime health‐related quality of life cost per case of bladder cancer was estimated as $428 689 (95% CI $427 753 to $438047), representing 65% of the total economic burden of this cancer.^20^ Similarly, the lifetime quality of life costs associated with each case of mesothelioma and lung cancer were estimated at $528 769 and $446 288 respectively, representing 67.6% of the total economic burden of these cancers.^6^

## 4 DISCUSSION

To the authors’ knowledge, this is the first systematic review to synthesize studies on the psychosocial cost burden associated with cancer. The review suggests that approximately 44% of individuals affected by cancer are impacted by psychosocial costs and that this burden has far reaching implications, significantly increasing the likelihood of poorer health, clinical, and economic outcomes for patients and caregivers. The estimated lifetime costs of the psychosocial burden were approximately CDN $428689 using conservative measures, representing two‐thirds of the total economic burden associated with a cancer diagnosis. This review highlights that this burden is likely too great to ignore, yet it remains a neglected topic in the published literature and potentially also in clinical settings.

Most studies in this review focused narrowly on a selection of constructs that related to the psychological and social domains of the psychosocial burden of cancer and, in particular, the financial impact of illness. This focus on financial impacts of cancer is likely a result of most research originating from the US where the impoverishing consequences of cancer are well documented.^37^ There was a lack of emphasis in studies investigating and capturing sub‐clinical levels of distress that are nevertheless significant and important for understanding the experience of psychosocial burden from patient, family, and caregiver perspectives. The findings of this review illustrate how little is yet known about the full spectrum of the psychosocial costs and their impact. However, that 44% of cancer patients were impacted by mostly financial consequences of cancer is concerning and aligns with findings from other studies.^32^, ^37^, ^38^

### 4.1 Clinical practice and research implications

This review confirms that more research on this topic is needed, particularly outside the United States. This review supports a call to action for a research agenda to investigate psychosocial costs more comprehensively in all settings given that we know that the psychosocial burden of cancer transcends borders.^9^ Within this agenda, there is also a need to better understand how this cost burden changes (or not) across the cancer treatment trajectory and into survivorship where the needs of cancer survivors change and are less well understood and less well met.^5^ This will inform the development of supports to better address the psychosocial burden of survivors.

This review also confirms that there is an inconsistent approach to the measurement of the psychosocial cost burden associated with cancer. Most studies used customized or purpose‐built measures, resulting in a range of tools in use in practice. In part, this may be due to the lack of a gold standard or validated approach for measurement. This has limited opportunities for comparison within and between cancer populations and across settings and this potentially hampers efforts to elevate the importance of this issue. Furthermore, it is well known that some sub‐populations are more at risk of facing an economic burden associated with cancer due to out‐of‐pocket,^35^, ^36^, ^39^ time,^40^, ^41^, ^42^ and indirect costs.^43^, ^44^, ^45^ A stronger commitment to consistently measure psychosocial costs, including in diverse cancer populations, is needed to determine who is most at risk and to help to direct action to address any inequities that may be contributing to such disparities in the economic burden of cancer.

The sole method used in the literature for monetizing the psychosocial costs was to calculate the cost per quality adjusted life year. Two generic tools, the SF‐12^46^ and the HUI,^47^ were used to measure health‐related quality of life. However, both tools do not explicitly capture elements relating to spiritual and social well‐being and thus they may not be sensitive enough to measure quality of life impacts related to these domains. This issue—the extent to which existing health‐related quality of life metrics (eg, quality‐adjusted life years) capture the breadth of psychosocial costs—has important implications for cancer care decision**‐**making. First, these existing tools potentially underestimate the psychosocial costs associated with cancer as they do not account for all dimensions of the psychosocial burden. Second, if these costs are to be included in economic evaluations of cancer interventions to better reflect the potential costs and benefits associated with cancer care, it is important to gain a better understanding of how to accurately capture and incorporate psychosocial costs to ensure they are not inadvertently double‐counted as both costs and benefits (eg, quality of life). Finally, the tendency in economic evaluations is to adopt a health care system perspective to best inform health system decision‐making.^48^ However, this review highlights the importance of adopting a broader societal perspective in economic evaluations to account for the significant psychosocial impact that is associated with cancer and borne by patients and their families. A recent systematic review of health interventions that included the patient perspective found no studies included intangible or psychosocial costs,^49^ further supporting that these costs are not routinely incorporated. Economic evaluations that account for the psychosocial burden associated with cancer should also be an input to support informed decision‐making, as this information will allow decision‐makers to weigh up the true value and cost‐effectiveness of new cancer interventions.

### 4.2 Study limitations

This study has limitations. We only included studies published in the last 10 years to ensure that, to the extent possible, the costs and policy contexts reflected contemporary circumstances. We also limited our inclusion criteria to English studies only. It is possible that we missed relevant studies; however, key experts were engaged to ensure seminal papers were not missed. The study quality of most studies was low. This limits the extent to which conclusions can be drawn but emphasizes the need for a research agenda to improve the measurement, breadth, and quality of studies on the psychosocial costs of cancer across settings.

## 5 CONCLUSION

The prevalence of the psychosocial cost burden found in this review supports a need for greater attention to address these costs, account for them in the context of economic evaluations and improve the psychosocial support programs available to cancer patients and their families. Elevating the importance of psychosocial costs is needed to drive efforts to develop more routine approaches to measurement as well as efforts to use these data to support decision making to lessen the economic burden faced by cancer patients and their families. Advancing our understanding of the psychosocial costs of cancer will help to meet commitments to patient‐centred care and research by improving our understanding of the full breadth of costs borne by patients and areas where supportive care can best mitigate this burden.

## ACKNOWLEDGEMENTS

This review was conducted by the Canadian Partnership Against Cancer (the Partnership). The Partnership is the steward of the Canadian Strategy on Cancer Control which provides a roadmap to improve equity in the cancer system and to deliver world‐class cancer care to everyone in Canada, while focusing on a sustainable healthcare system for the future. The results of this review will inform efforts to strengthen supports available to cancer patients, aligned with the key priorities and actions outlined in the Strategy.

## Supporting information


**Figure S1** Risk of bias

**Data S1**. Supporting Information.

## Data Availability

The data that support the findings of this study are available from the corresponding author upon reasonable request.
