# Supplementary figures and images for: The psychosocial cost burden of cancer: A systematic literature review
Source: Psychooncology. 2020 Sep 6;29(11):1746–60. doi: 10.1002/pon.5516 (PMC7754376; doi:10.1002/pon.5516)

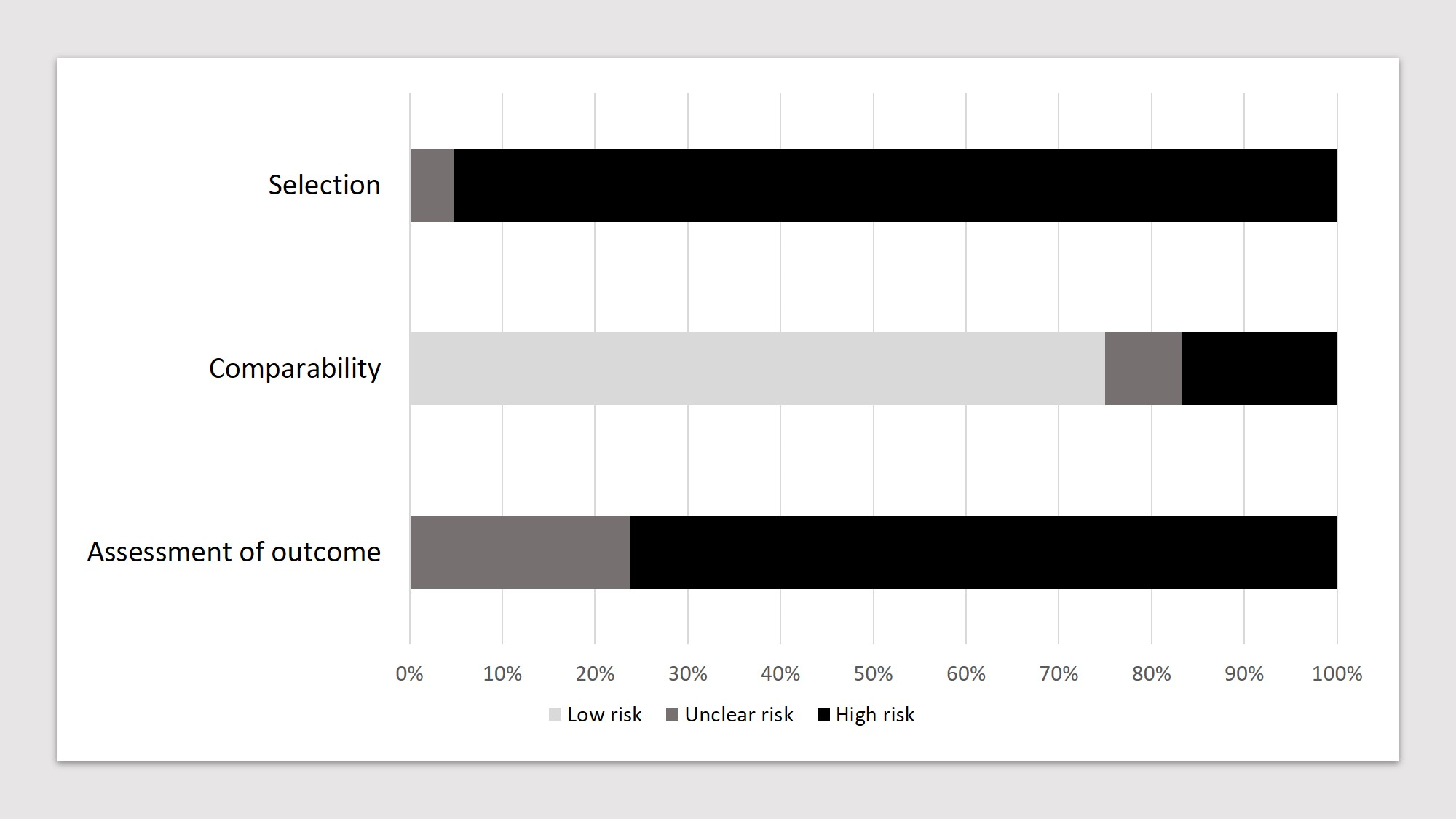

Supplement: Supplementary file 1 — Figure S1 Risk of bias [file PON-29-1746-s001.png]
