# Supplementary material for: The psychosocial cost burden of cancer: A systematic literature review
Source: Psychooncology. 2020 Sep 6;29(11):1746–60. doi: 10.1002/pon.5516 (PMC7754376; doi:10.1002/pon.5516)
Supplement: Supplementary file 2 — Data S1. Supporting Information. [file PON-29-1746-s002.docx]

The psychosocial cost burden of cancer: A systematic literature review

Online supplement

# Protocol and search strategy

**Databases searched:**

Pubmed, Medline, Embase, Cinahl, PsycInfo, Econlit, Johanna Briggs EBP database.

Google Scholar, OpenGrey and Grey Literature Report

**Search questions:**

1. What is the psychosocial cost burden associated with cancer and how do studies measure psychosocial costs?
2. What is the prevalence and impact of this burden?

*Population:* All cancers, all patients

*Intervention:*  none

*Comparator:* No cancer if available, otherwise no comparator

*Outcome:* Psychosocial costs, however defined

**Inclusion criteria:**

- Defines ‘psychosocial costs’ explicitly or in terms of the factors that contribute to the psychosocial cost burden
- Psychosocial costs experienced by patients along the cancer care trajectory (diagnosis to end of life care)

**Exclusion criteria:**

- Date: Study conducted prior 2008
- Language: not English language
- Full-text: article unavailable

**Quality review:**

Ottawa-Newcastle risk of bias assessment tool: observational studies

**Data extraction:**

- Double- data extraction
- Extract the following information:
  - Country
  - Year
  - Study population
    - Adults/ children
    - Cancer type
    - Where on the cancer care continuum
    - Sample size
  - Setting
  - Design
  - Data collection / data source
    - Name of tool used for measurement
      - Validated?
    - Time frame
  - Dimension of psychosocial cost measured
  - Estimate of psychosocial cost or burden
  - Key findings
- Extract information for all psychosocial costs estimates associated with cancer at any point along the care trajectory (diagnosis to end of life) and for people with cancer, family members and/ or caregivers

**Search strategy: Medline**

*Population:* All cancers, all patients

Neoplasm*.ti. OR Cancer*.ti. OR “cancer survivors” .ti.

*Outcome:* Psychosocial costs

(Psycho*.ti OR anxiety.ti OR depression.ti OR (cognitive and impairment).ti OR (mental and illness.ti OR (emotional and health.)ti OR (mental and health).ti OR (psycho* and health).ti OR (psycho* and distress).ti OR stress.ti OR mood.ti OR pain.ti OR suffer*.ti OR grief.ti OR fear*.ti OR bereavement.ti OR worr*.ti OR sorrow.ti OR spirit*.ti OR hope*.ti OR quality of life[MESH] OR fatigue.ti OR despair.ti OR distress.ti OR cope.ti OR coping.ti. (economic and burden).ti OR (economic and impact).ti OR (financial and burden).ti OR (financial and impact).ti OR (financial and toxicity).ti OR (financial and stress).ti

AND

cost* OR burden* OR impact*
